# Interplay between human microglia and neural stem/progenitor cells in an allogeneic co-culture model

**DOI:** 10.1111/jcmm.12123

**Published:** 2013-09-12

**Authors:** Jia Liu, Erik Hjorth, Mingqin Zhu, Cinzia Calzarossa, Eva-Britt Samuelsson, Marianne Schultzberg, Elisabet Åkesson

**Affiliations:** a Department of Neurobiology, Care Sciences & Society Karolinska Institutet, Geriatric Clinic Res LabStockholm, Sweden; b Department of Neurology, First Hospital of Jilin UniversityChangchun, China; c Stockholms Sjukhem Foundation, R&D DepartmentStockholm, Sweden

**Keywords:** CD200, human neural stem/progenitor cells, immunomodulation, microglia, phagocytosis, M1/M2 phenotype, transforming growth factor-β

## Abstract

Experimental neural cell therapies, including donor neural stem/progenitor cells (NPCs) have been reported to offer beneficial effects on the recovery after an injury and to counteract inflammatory and degenerative processes in the central nervous system (CNS). The interplay between donor neural cells and the host CNS still to a large degree remains unclear, in particular in human allogeneic conditions. Here, we focused our studies on the interaction of human NPCs and microglia utilizing a co-culture model. In co-cultures, both NPCs and microglia showed increased survival and proliferation compared with mono-cultures. In the presence of microglia, a larger subpopulation of NPCs expressed the progenitor cell marker nestin, whereas a smaller group of NPCs expressed the neural markers polysialylated neural cell adhesion molecule, A2B5 and glial fibrillary acidic protein compared with NPC mono-cultures. Microglia thus hindered differentiation of NPCs. The presence of human NPCs increased microglial phagocytosis of latex beads. Furthermore, we observed that the expression of CD200 molecules on NPCs and the CD200 receptor protein on microglia was enhanced in co-cultures, whereas the release of transforming growth factor-β was increased suggesting anti-inflammatory features of the co-cultures. To conclude, the interplay between human allogeneic NPCs and microglia, significantly affected their respective proliferation and phenotype. Neural cell therapy including human donor NPCs may in addition to offering cell replacement, modulate host microglial phenotypes and functions to benefit neuroprotection and repair.

## Introduction

Neurodegenerative disorders as well as trauma to the central nervous system (CNS) result in damage and loss of neural cells, as well as inflammation. Within the CNS, microglia are known as the primary mediators of the innate immune response. Under normal conditions, they play an important role in immune surveillance and homeostasis 1. In response to a range of stimuli associated with pathological changes, microglia are rapidly activated, and their random surveilling movements become selectively directed to the injured site 2. Microglia can play either a neurotoxic or neuroprotective role. The neurotoxic activities are usually associated with the ‘classical activation’ (M1) phenotype, and their release of pro-inflammatory cytokines, such as tumour necrosis factor (TNF)-α and interleukin (IL)-1, contributing to neuronal dysfunction and cell death 3. Whereas the protective activities are associated with the ‘alternative activation’ (M2) phenotype, and their production of neuroprotective substances, such as anti-inflammatory cytokines and neurotrophic factors, promoting tissue repair by blocking the production of reactive oxygen intermediates and pro-inflammatory cytokines 4.

Cell therapy including human neural stem/progenitor cells (NPCs) derived from embryonic stem cells, foetal CNS or induced pluripotent stem cells, is considered as a promising therapeutic approach to protect and restore the damaged CNS. Beneficial effects including improvements of motor or cognitive functions have been demonstrated in animal models of Parkinson's disease 5, spinal cord injury 6 and Alzheimer's disease (AD) 7 after neural cell therapy.

The microglial activation states have been shown to affect NPC differentiation in animal models in different ways 8,9. In turn, NPCs may affect immune cells. Cusimano *et al*. 11 showed that transplanted mouse NPCs reduced the proportion of M1 macrophages, leading to a reprogramming of the local inflammatory cell microenvironment from a ‘hostile’ to an ‘instructive’ state, thus promoting the healing of the injured spinal cord. Recently, Mosher *et al*. 12 reported that both mouse and rat NPCs regulated microglial functions and activity. However, the interplay of human NPCs and microglial cells remains, still to a large degree, unclear.

In this study, we utilized a human allogeneic co-culture model to evaluate reciprocal effects of human NPCs and microglia. With the aim to model a naive encounter of donor human NPCs with human microglia, we seeded ‘non-activated’ microglial cells with NPCs early during differentiation. We analysed the differentiation profile of NPCs, the phenotype polarization of microglia and the potential interaction between NPCs and microglia, all of importance to understand when designing future neural cell therapies.

## Materials and methods

### Culture of human foetal neural stem/progenitor cells

Cultures of NPCs were derived from human first trimester CNS tissue, 5–7.5 weeks of gestation. The procedure was approved by the Regional Ethics Committee, Stockholm, Sweden. Briefly, CNS tissue was retrieved from clinical first trimester elective abortions after informed consent. Isolated spinal cord tissue was homogenized and cultured in NPC medium (DMEM/F12, 0.6% glucose, 5 mM Hepes, 2 μg/ml Heparin, 1% N_2_ supplement, all from Life Technologies, Carlsbad, CA, USA) supplemented with 20 ng/ml human epidermal growth factor, 20 ng/ml human basic fibroblast growth factor and 10 ng/ml ciliary neurotrophic factor (all from R&D, Minneapolis, MN, USA) as previously described 13. The cultures of free-floating neurospheres were passaged every 7–14 days by enzymatic dissociation using TrypLE Express (Life Technologies) and gentle trituration with fresh medium. All cultures were maintained in a humidified atmosphere at 37°C and 5% CO_2_ with fresh medium added twice a week. Human neurospheres (including NPCs) between passages 5 and 10 were used in the following experiments.

### Culture of human microglia

The human microglia line CHME3 was obtained as a kind gift from Professor M. Tardieu (Neurologie pédiatrique, Hôpital Bicêtre, Assistance publique, Hôpitaux de Paris, Paris, France), and cultured as described previously 14. Briefly, CHME3 microglial cells (hereafter called microglia) were cultured in flasks in culture medium [DMEM/high glucose w/o sodium pyruvate supplemented with 2 mM GlutaMaxII and 10% heat-inactivated foetal bovine serum (FBS), all from Life Technologies] at 37°C and 5% CO_2_. The cells were subcultured at confluence using enzyme-free cell dissociation buffer (Life Technologies).

### Co-cultures of NPCs and microglia

**Figure 1:**
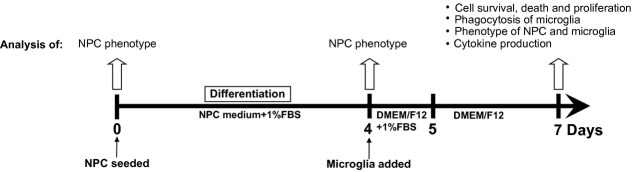
Schematic illustration of the experimental design.

The experimental design is shown in Figure 1. Briefly, human neurospheres including NPCs were dissociated into single-cell suspension and seeded onto poly-d-lysine (0.1 mg/ml, Sigma-Aldrich, St. Louis, MO, USA) and fibronectin (0.1 mg/ml, Sigma-Aldrich) coated plates or flasks at a concentration of 10,000 cells/cm^2^. Human NPCs were differentiated in NPC medium with the presence of 1% FBS (without mitogens present) for 4 days at 37°C and 5% CO_2_ to achieve a neural progenitor cell population just entering differentiation. To mimic an encounter of naive host microglial cells with donor NPCs, unstimulated microglia were added directly to the cultures of differentiating NPCs on day 4, allowing cell–cell contact at a concentration of 10,000 cells/cm^2^ and the medium volume of 0.1 ml/cm^2^. Co-cultures were kept in DMEM/F12 and 1% FBS for 24 hrs to allow microglial cells to attach. Thereafter, NPCs and microglia were co-cultured in serum-free medium for up to 48 hrs to avoid the interference of serum to the subsequent cytokine analysis. NPC and microglial mono-cultures were processed as controls. All cultures were kept under consistent and equivalent conditions. NPCs co-cultured, under equivalent conditions in sequence as above, with equal amount of human foreskin fibroblasts (hereafter called fibroblasts) or NPCs were also set up as controls for some experiments. On day 7, the cells were harvested and further analysed as described below.

### Cell viability and death

To assess cell viability, the culture medium was removed and cultures were washed with serum- and phenol-free medium. The resazurin sodium salt (20 μg/ml, Sigma-Aldrich), used as an indicator of cell metabolic activity, was added and the cells incubated at 37°C for 2 hrs. Wells containing only medium and resazurin were used as blanks. The fluorescence of formazan salt was measured with a TECAN Safire2 microplate reader (Männedorf, Switzerland) at 590 nm. Results are presented as differences in fluorescence intensity (fluorescence_sample_ – fluorescence_blank_).

Cell death was evaluated utilizing the necrosis marker 7-amino-actinomycin (7-AAD) and intracellular apoptosis marker active caspase-3 by flow cytometry. In co-cultures, NPCs or microglia were labelled with 5,6-carboxyfluorescein diacetate succinimidyl ester (CFSE, Life Technologies) to distinguish different cell populations (see below).

### Proliferation assay

Carboxyfluorescein diacetate succinimidyl ester-based proliferation assay, depending on a reduction of CFSE fluorescence intensity in successive generations of a proliferating cell population, was performed to evaluate the degree of cell proliferation. Briefly, before start of co-culture, CFSE [5 μM, diluted in warm PBS, 0.1% human serum albumin (Sigma-Aldrich)] was added to NPCs or microglia and left to penetrate into cell membranes for 15 min. at 37°C, followed by 30 min. incubation at 37°C with fresh culture medium to remove excess reagent. Cells were co-cultured for 3 days, and then the percentage of divided cells was analysed by flow cytometry.

### Phagocytosis assay

To analyse the microglial phagocytic activity, microglial cells were labelled by fluorescent probe 5-(and 6)-(((4-chloromethyl)-benzoyl) amino)-tetramethyl-rhodamine (CMTMR, 10 μM, Life Technologies). On day 7, cells were incubated with fluorescent yellow-green latex beads (diameter = 0.03 μm, 1:1000, Sigma-Aldrich) at 37°C for 2 hrs. Microglial mono-cultures were set up as control. Afterwards, cells were harvested and microglial uptake of latex beads was analysed by flow cytometry.

### Flow cytometry

Details are provided in Supplementary materials and Table S1.

### Immunocytochemistry

The indirect immunocytochemistry procedure was performed as described previously 13. The z-stack confocal images were acquired using an inverted microscope Axiovert 200M (Carl Zeiss MicroImaging GmbH, Jena, Germany) connected to a LSM510 META confocal unit. Further details are provided in Supplementary materials and Table S2.

### ELISA

The release levels of TNF-α, IL-6, IL-10, transforming growth factor (TGF)-β1, TGF-β2, brain-derived neurotrophic factor (BDNF), glial cell line-derived neurotrophic factor (GDNF), neurotrohpin-3 (NT-3) in the culture media were measured by ELISA. Details are provided in Supplementary materials.

### Statistical analysis

Analysis of differences among groups was performed by either parametric or non-parametric tests (depending on whether the data were normally distributed or not), using the Instat 3.0 software for Macintosh (GraphPad Software Inc., La Jolla, CA, USA). The statistical method applied is indicated in each figure legend. *P*-values less than 0.05 were considered statistically significant. The mean values ± SEM or median ± percentiles (25–75% and 5–95%) are presented in the figures. The ‘n’ indicated in each figure legend refer to number of independent human NPC cases tested per group.

## Results

### Increased cell survival in NPC and microglial co-cultures

**Figure 2:**
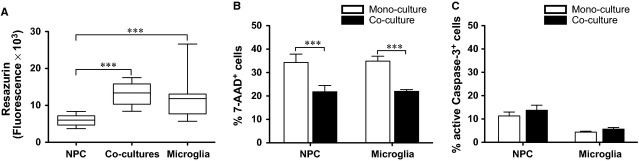
Reduced necrotic cell death in neural stem/progenitor cell (NPC) and microglial co-cultures. (A) Cell viability as determined by resazurin conversion assay is presented as fluorescence equivalent to cell number at the end-point of co-cultures. The differences among groups (*n *=* *5/group) were analysed by Kruskal–Wallis test with Dunn's *post hoc* test. Median ± percentiles (25–75% and 5–95%). Cell death was evaluated by the application of (B) the necrosis marker 7-amino-actinomycin and (C) the intracellular apoptosis marker active caspase-3, shown as the percentage of immunolabeled cells as evaluated by flow cytometry (*n *=* *6/group) and analysed by *t*-test. Mean values ± SEM. ***P < 0.001.

The survival of NPCs and microglia was evaluated on day 7 in mono- and co-cultures. Ocular inspection of the cultures under phase contrast microscope on day 7 revealed a relatively stable overall viability (Fig. S1). Cell viability was further analysed by resazurin conversion assay. Co-cultures and microglial mono-cultures showed a significantly higher metabolic activity compared with NPC mono-cultures (Fig. 2A, *P* < 0.001). Cell death was investigated by analysing necrosis (7-AAD) and apoptosis (active caspase-3) markers. A smaller population of 7-AAD^+^ NPCs and microglia was observed in the co-cultures compared with their respective mono-cultures (Fig. 2B, *P* < 0.001). In contrast, the subpopulation expressing intracellular active caspase-3 was unaffected in size between mono- and co-cultures (Fig. 2C). When human NPCs were cultured either with themselves at higher density with the double amount of cells or in co-culture with fibroblasts as additional controls, the percentage of 7-AAD^+^ NPCs did not significantly differ from regular NPC mono-culture (Fig. S2A). This suggests that the increased NPC survival in co-cultures was due to the presence of microglia rather than a higher cell density *per se*.

### Microglia promoted NPC proliferation, while hindering NPC differentiation

**Figure 3:**
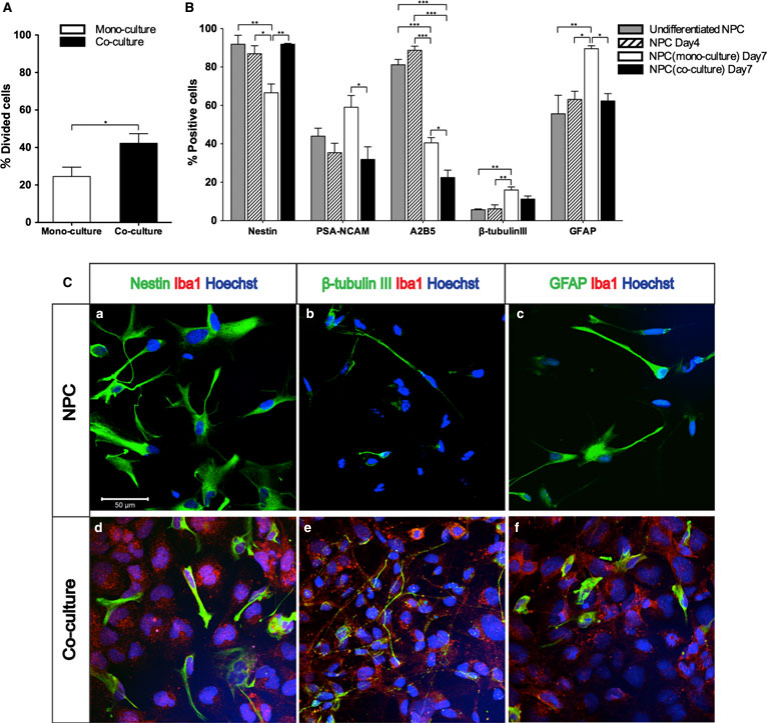
Microglia promoted neural stem/progenitor cell (NPC) proliferation, while reducing NPC differentiation. The effects of the presence of microglia on NPCs were studied in mono- and co-cultures. (A) Proliferation of NPCs (*n *=* *5/group) was analysed using carboxyfluorescein diacetate succinimidyl ester-based proliferation assay by flow cytometry and presented as percentage of divided cells out of total NPC population. Differences were evaluated by Mann–Whitney test. (B) Flow cytometry analysis of NPC phenotype in the absence or presence of microglia. The percentage of cells immunoreactive for nestin, polysialylated neural cell adhesion molecule, A2B5, β-tubulin III and glial fibrillary acidic protein (GFAP), out of the total NPC populations (*n *=* *7/group), was evaluated by anova with Turkey *post hoc* test. NPCs prior to differentiation and NPCs differentiated for 4 days before addition of microglia served as controls. Mean values ± SEM. *P < 0.05, **P < 0.01, ***P < 0.001. (C) Representative confocal microphotographs of NPCs in mono- and co-cultures at day 7, immunolabeled with nestin (a, d), β-tubulin III (b, e) and GFAP (c, f) are shown. Nuclei are labelled in blue (Hoechst). Scale bar = 50 μm.

Neural stem/progenitor cell proliferation was analysed by CFSE-based proliferation assay and presented as percentage of divided cells out of total NPC population. In co-cultures, the percentage of NPCs undergoing cell division was significantly increased compared with NPC mono-cultures (Fig. 3A, *P* < 0.05), suggesting that the presence of microglia promoted NPC proliferation.

Analysis of additional control cultures prior to differentiation showed that ∼92% of NPCs were nestin^+^, whereas 44, 81 and 56% of total cell numbers were polysialylated neural cell adhesion molecule (PSA-NCAM)^+^, A2B5^+^ or glial fibrillary acidic protein (GFAP)^+^, respectively. In contrast, only 6% of NPCs were β-tubulin III^+^ (Fig. 3B). After 7 days in differentiation medium, the percentage of nestin^+^ and A2B5^+^ cells decreased in NPC mono-cultures, whereas the subpopulation of cells expressing β-tubulin III and GFAP was significantly increased (Fig. 3B, C-a, b, c, *P* < 0.01).

When microglia were added to the differentiating NPC cultures (Fig. 3B, C-d, e, f), there were no significant changes in the size of nestin^+^, PSA-NCAM^+^, β-tubulin III^+^ or GFAP^+^ NPC subpopulations compared with undifferentiated NPCs (Fig. 3B); whereas the percentage of A2B5^+^ cells significantly decreased compared with undifferentiated NPCs (Fig. 3B, *P* < 0.001). On the other hand, the subpopulations of PSA-NCAM^+^, A2B5^+^ or GFAP^+^ cells in NPCs co-cultured with microglia were significantly smaller than in the cultures with NPCs differentiated alone (Fig. 3B, *P* < 0.05); whereas the proportion of nestin^+^ cells was higher in the co-cultures compared to that in the NPC mono-cultures (Fig. 3B, *P* < 0.001). When cultured in double numbers as mono-cultures or in co-culture with fibroblasts, the expression of neural markers on NPCs was not statistically different from that detected in the regular NPC mono-cultures (Fig. S2B), suggesting that alterations of NPC phenotype were here specific for co-cultures and interactions with microglia.

### NPCs enhanced microglial proliferation, phagocytic activity and affected microglial phenotype

**Figure 4:**
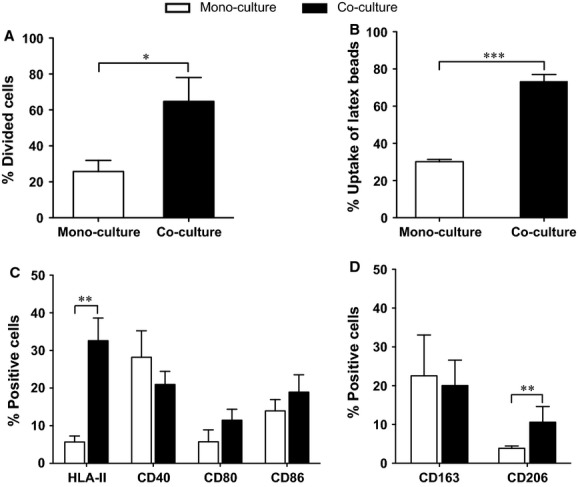
Neural stem/progenitor cells (NPCs) affected microglial activity and phenotype. The effects of NPCs on the microglia were studied in co-cultures with mono-cultures as control. (A) Proliferation of microglia (*n *=* *5/group) was analysed by carboxyfluorescein diacetate succinimidyl ester-based proliferation assay and presented as percentage of divided cells of the total microglial population. Differences between mono- and co-cultures were evaluated by Mann–Whitney test. (B) Fluorescent latex beads were added to the cultures for 2 hrs, and the uptake of beads by microglia was evaluated by flow cytometry. Percentage of microglial cells with latex beads out of total microglial population is presented and statistically analysed by *t*-test between mono- and co-cultures. Expression of (C) M1-markers: HLA-II and co-stimulatory molecules (CD40, CD80 and CD86), and (D) M2-markers: CD163 and CD206 on microglia (*n *=* *7/group), were evaluated by flow cytometry and shown as percentage of positive cells out of total cell number. The data were analysed by Mann–Whitney test between mono- and co-cultures. Mean values ± SEM. *P < 0.05, **P < 0.01, ***P < 0.001.

In co-cultures, the population of divided microglia was significantly larger than that in microglial mono-cultures (Fig. 4A, *P* < 0.05), suggesting that presence of NPCs promoted microglial proliferation.

To investigate whether NPCs affect microglial phagocytic activity, fluorescent latex beads were added to the cultures. The uptake of latex beads by microglia was confirmed by confocal analysis (Fig. S3) and further quantified by flow cytometry. In co-cultures, the percentage of microglia phagocyted latex beads was significantly higher than in mono-cultures (Fig. 4B, *P* < 0.001). In addition, the mean fluorescence intensity (MFI) of latex beads in co-cultures was significantly higher compared with microglial mono-cultures (268 ± 17 *versus* 200 ± 12, *P* = 0.0006), indicating that microglia engulfed more latex beads in co-cultures than in mono-cultures.

The expression of M1-markers (HLA-II, CD40, CD80 and CD86) and M2-markers (CD163 and CD206) on microglia was analysed by flow cytometry. In the presence of NPCs, the subpopulation of microglia expressing HLA-II was significantly higher (33%) than in mono-cultures (6%) (Fig. 4C, *P* < 0.01). Furthermore, the MFI was above 100 in co-cultures and below 55 in mono-cultures confirming the up-regulation of HLA-II expression. The percentage of CD206^+^ microglia out of the total microglial population in co-cultures was clearly higher than that in mono-cultures (Fig. 4D, *P* < 0.01), whereas the MFI was stable above 150 on microglia in both mono- and co-cultures. The expression of the co-stimulatory molecules CD40, CD80 and CD86, and the scavenger receptor CD163 on microglia did not differ significantly between co- and mono-cultures (Fig. 4C and D).

### Increased CD200 and CD200R immunoreactivity in NPC and microglial co-cultures

**Figure 5:**
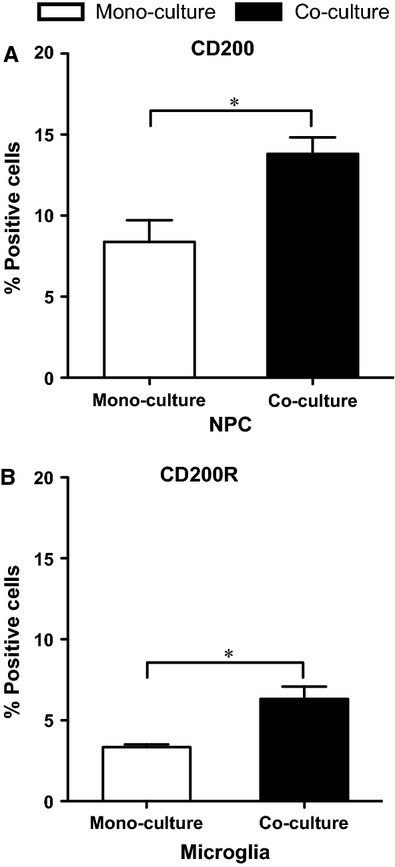
Enhanced expression of CD200 and CD200R on neural stem/progenitor cells (NPCs) and microglia in co-cultures. The expression of (A) CD200 on NPCs and (B) CD200R on microglia (*n *=* *6/group) was assessed by flow cytometry. The data are shown as percentage of positive cells out of total cell number of respective cell type and analysed by *t*-test between mono- and co-cultures. Mean values ± SEM. *P < 0.05.

We further studied the presence of the neuroimmune regulatory protein CD200 and its receptor, CD200R, on NPCs and microglia, respectively. The percentage of CD200^+^ NPCs was significantly increased from 8% in mono-cultures to 14% in co-cultures (Fig. 5A, *P* < 0.05). At the same time, the proportion of microglial cells expressing CD200R out of the total microglia population in the co-cultures was significantly higher than that in microglial mono-cultures (Fig. 5B, *P* < 0.05).

### Cytokines in NPC and microglial co-cultures

**Figure 6:**
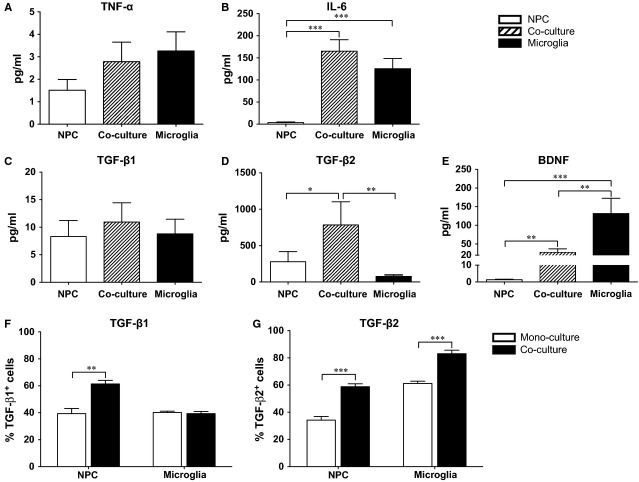
Cytokine production in neural stem/progenitor cell (NPC) and microglia cultures. A total amount of 10 ml culture medium from mono- and co-cultures (*n *≥* *6/group) was collected and the levels of the cytokines/neurotrophic factor (A) tumour necrosis factor-α and (B) IL-6, (C) transforming growth factor (TGF) -β1, (D) TGF-β2 and (E) brain-derived neurotrophic factor, were measured by ELISA. The data were analysed by Kruskal–Wallis test with Dunn's *post hoc* test. The expression of (F) TGF-β1 and (G) TGF-β2 in NPCs and microglia was further assessed by flow cytometry, and the data were analysed by *t*-test between mono- and co-cultures. Mean values ± SEM. *P < 0.05, **P < 0.01, ***P < 0.001.

The release of IL-6 was undetectable in NPC mono-cultures, whereas co-cultures and microglial mono-cultures resulted in higher levels (Fig. 6B). In the medium of co-cultures, the levels of TGF-β2 were significantly higher than that observed in the NPC and microglial mono-cultures, respectively (Fig. 6D, *P* < 0.05). In addition, we observed that the BDNF concentration in the co-culture supernatant was significantly lower compared with microglial mono-cultures, but higher than in NPC mono-cultures (Fig. 6E, *P* < 0.01). The release of TNF-α (Fig. 6A), TGF- β1 (Fig. 6C), IL-10, GDNF and NT-3 (data not shown) in co-cultures did not differ significantly from mono-cultures of either microglia or NPCs. To rule out background signals, we analysed the growth medium alone and found undetectable levels of these studied cytokines.

Finally, the proportion of cells, in the respective cell culture type, expressing TGF-β1 or TGF-β2 was evaluated by flow cytometry. The proportion of TGF-β1^+^ NPCs was significantly higher in the presence of microglia as compared with NPC mono-cultures (Fig. 6F, *P* < 0.01), whereas no difference was observed between mono- and co-cultures in microglia. In co-cultures, both NPCs and microglia presented higher percentages of TGF-β2^+^ cells compared to that in their respective mono-cultures (Fig. 6G, *P* < 0.001).

## Discussion

The interaction of NPCs with immune cells is of great interest both related to the understanding of neuropathological mechanisms, endogenous neurogenesis and treatment options, including neural cell therapy, in neurotrauma and neurodegenerative disease. We have previously studied human NPCs and their interaction with human lymphocyte populations 15. In this study, we utilized a human allogeneic co-culture model to study the potential interplay between NPCs and a microglial cell population. The aim was to mimic the naive encounter between human ‘host’ microglial cells with a ‘donor’ NPC population during early differentiation as a model of relevance for neural cell therapy development. We report that the presence of microglia enhanced proliferation, but hindered differentiation of NPCs, whereas NPCs increased proliferation and phagocytic activity as well as influenced the phenotype of the microglia.

Co-culture with microglia increased the proliferation of NPCs significantly, which is in accordance with rodent studies by Cacci *et al*. 9 and Mosher *et al*. 12, where conditioned medium from untreated microglia were added to NPCs. Activated microglia from injured brains of rodents also increased the proliferation of NPCs 16, while Taylor *et al*. 17 demonstrated that conditioned medium from activated rodent microglia attenuated proliferation in a primary oligodendrocyte precursor cell population. Furthermore, Gu *et al*. 8 showed that untreated microglia promoted astrogliogenesis from NPCs. In this study, human NPCs in co-cultures with human microglia presented a higher percentage of nestin, and a lower proportion of PSA-NCAM, A2B5 and GFAP immunoreactive cells compared with mono-cultures. This suggests that the presence of microglia inhibited NPC maturation and differentiation with regard to both neuronal or astroglial destiny. These observations could be partially explained by the presence of IL-6 released by microglia in the co-cultures as suggested by other research groups 18. Our data, from human allogeneic co-cultures, confirm others’ findings, indicating that the microglial cell population can act as a determinant of NPC differentiation.

Microglia, the resident antigen-presenting cells of the CNS, are known to be highly plastic cells displaying diverse reactions associated with both deleterious and protective effects on the surrounding neural parenchyma upon activation 19. Mosher *et al*. 12 reported that both mouse and rat NPCs can increase the activation, proliferation and phagocytosis of mouse microglia. In agreement, we found that the proliferation and phagocytic activity of human microglia were significantly increased when co-cultured with human NPCs. Also, the proportion of microglia positive for M1-marker HLA-II or M2-marker CD206 was significantly increased in this study. These data suggest that NPCs can influence the degree of microglial activation and functional activity *in vitro*, and that different microglial phenotype can emerge during these human co-culture conditions.

To counteract neurotoxic effects of innate immune molecules, both glia and neurons express a range of neuroimmune regulatory proteins (NIRegs), including CD200 20–21. Neuroimmune regulatory proteins in analogy to regulatory T cells (Tregs), are involved in silencing and reshaping an adverse innate immune response, and in polarizing microglia towards a protective phenotype, to limit inflammatory reactions to the injury site 22. CD200R (mainly expressed on cells of myeloid origin, such as microglia) is structurally closely related to CD200, but has a longer cytoplasmic tail that delivers an inhibitory signal after ligation to CD200 23. The CD200-CD200R interaction provides a negative cell–cell contact dependent regulatory signal for microglia. This interaction was reported to down-regulate inflammation in diseases such as multiple sclerosis 24 and AD 25. Deficit in the CD200-CD200R system has been shown to be impaired in AD 25 and may exacerbate neurodegeneration in a model of Parkinson's disease 26. In addition, increased neuronal levels of CD200 may protect mice from inflammation-mediated neurodegeneration 27. Therefore, it is reasonable to assume that a CD200–CD200R interaction plays an important role in regulating microglial activity and limiting neuroinflammation. In the present human co-culture system, the conditions provides ample opportunity of cell–cell interaction that are lacking in studies performed with conditioned microglial medium alone. We showed that not only did a larger proportion of NPCs express CD200 but also the expression of CD200R on microglia was up-regulated in the co-cultures. As a result, the opportunity for a CD200–CD200R interaction between the human NPCs and microglia was enhanced, which may limit deleterious neuroinflammation caused by microglia.

Several studies have demonstrated that blocking the CD200–CD200R interaction can lead to decreased TGF-β expression 28–29, in other words CD200–CD200R interaction may support the release of TGF-β. In this study, the secretion of TGF-β2 by both NPCs and microglia was higher in co-cultures compared with mono-cultures. In addition, the subpopulation of TGF-β1^+^ NPCs increased, which could be explained by the enhanced CD200–CD200R interaction. TGF-β is known to enhance the survival of phagocytic microglia 30, and to increase the uptake of AD-related β-amyloid (Aβ) peptide by microglia 31. In our model, the enhanced phagocytic activity of microglia in co-cultures may be related to the increased release of TGF-β. TGF-β2 may also help to polarize microglia towards an anti-inflammatory phenotype, and to influence the antigen-presenting functions of microglia 32–33. We observed that in co-cultures, the expression of the anti-inflammatory M2-marker CD206 was up-regulated, but not the co-stimulatory molecules, which are related to the antigen-presenting ability of microglia. We have previously shown that microglia engaging in phagocytosis of Aβ expressed CD206 to a larger degree than the non-phagocytic microglia 34, adding credibility to the opinion that CD206 is associated with beneficial and neuroprotective microglial activities.

The levels of BDNF decreased upon co-culture of NPCs and microglia compared with the microglial mono-cultures. BDNF, which in our model appears to be mostly of microglial origin, is an important survival and differentiation factor for neuronal cells 35. Suppression of BDNF secretion, if occurring in the *in vivo* situation, can be deleterious for neurons in a damaged area. However, the data on BDNF must be regarded as part of the whole picture of our results, which show a general pro-survival and anti-inflammatory effect of the NPC-microglia interaction. Our findings based on studies of initially non-activated microglia in this human allogeneic model provide a groundwork for further studies to be performed with microglia under different activation states resembling the situation in lesion and disease of the spinal cord and brain, including AD.

In summary, in an allogeneic human co-culture model, both NPCs and microglia were significantly affected by each other's presence. The observed reciprocal influence suggests a human NPC and microglial interplay regulating neural proliferation as well as microglial phagocytic activity, phenotype polarization and cytokine production. These findings support a neuroprotective role of human neural cell therapy including NPCs, in addition to offering a potential cell replacement therapy.

## Supporting Information

Figure S1 Representative phase contrast microphotographs of mono- and co-cultures at day 7 are shown. Scale bar = 200 μm.

Figure S2 Flow cytometry analysis of human NPC death and phenotype in the cultures with higher density of NPCs or co-cultures of NPCs and human foreskin fibroblast. The percentage of cells immunoreactive for (A) 7-AAD, (B) nestin, PSA-NCAM, A2B5, β-tubulin III and GFAP out of the total NPC populations (n ≥ 4/group) was evaluated by ANOVA with Turkey post hoc test. Mean values ± SEM.

Figure S3 Representative confocal microphotograph of fluorescent latex beads (green) in microglia at day 7 is shown. Nuclei are labelled in blue (Hoechst). Scale bar = 20 μm.

**Table S1** Antibodies used in flow cytometry. **Table S2** Antibodies used in immunocytochemistry.
