# Supplementary figures and images for: Interplay between human microglia and neural stem/progenitor cells in an allogeneic co-culture model
Source: J Cell Mol Med. 2013 Sep 12;17(11):1434–43. doi: 10.1111/jcmm.12123 (PMC4117556; doi:10.1111/jcmm.12123)

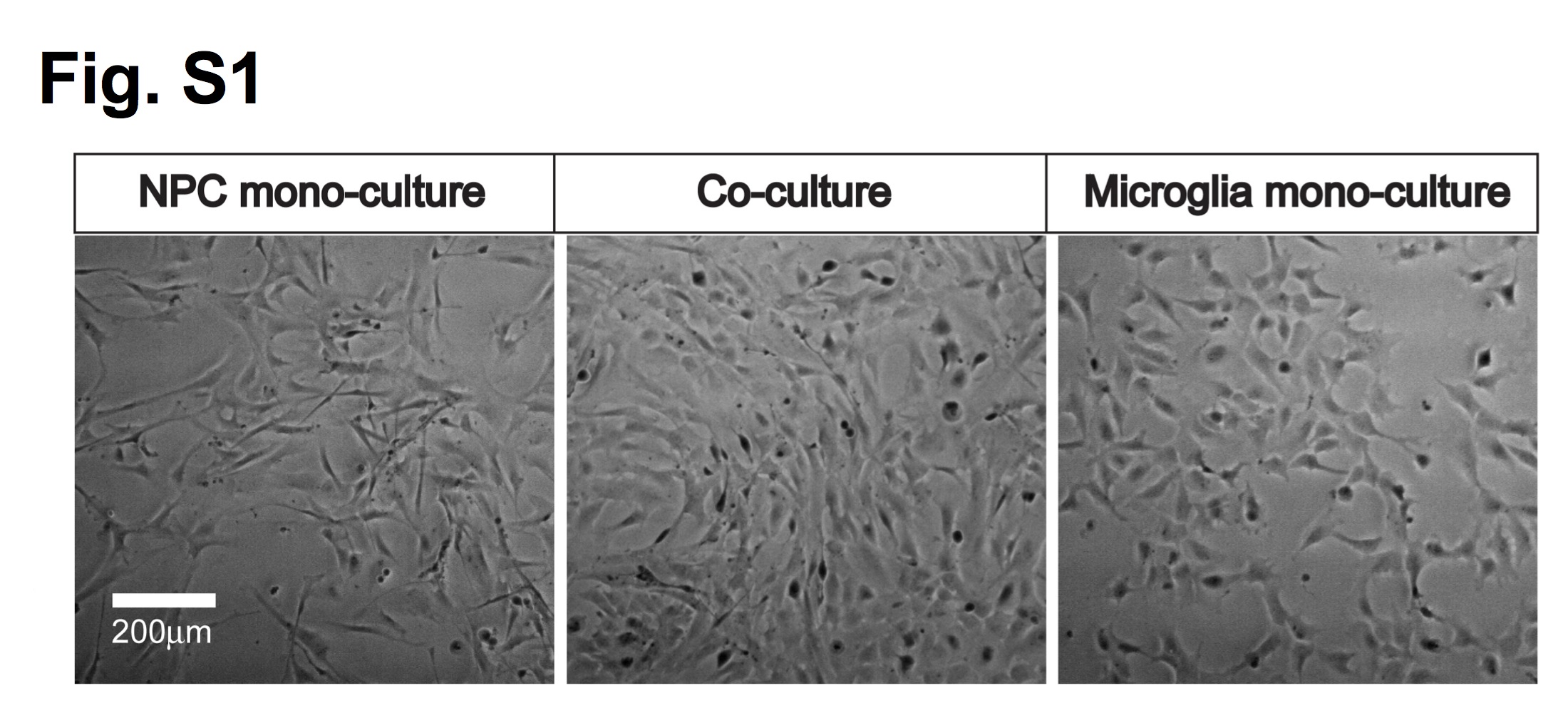

Supplement: Figure S1 — Representative phase contrast microphotographs of mono- and co-cultures at day 7 are shown. Scale bar = 200 μm. [file jcmm0017-1434-sd1.jpg]

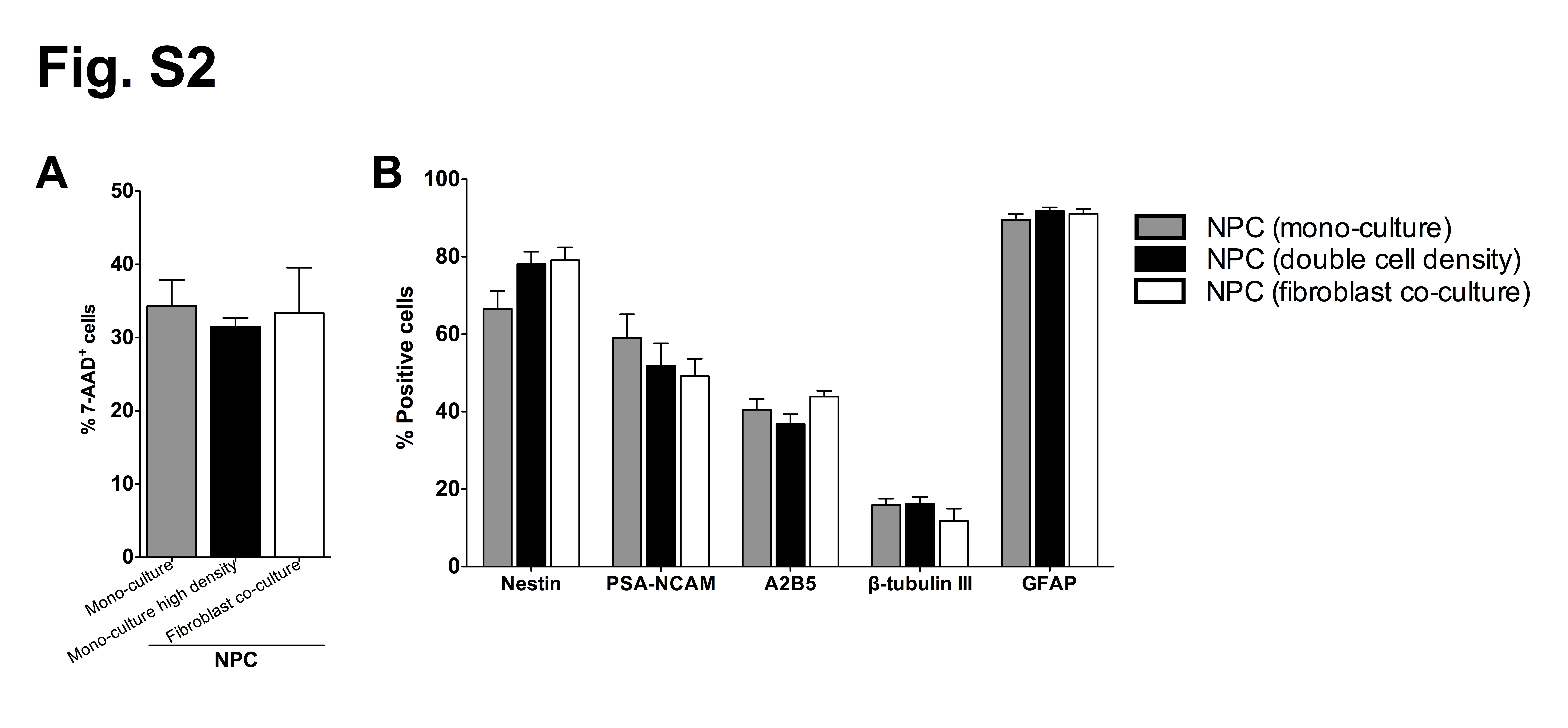

Supplement: Figure S2 — Flow cytometry analysis of human NPC death and phenotype in the cultures with higher density of NPCs or co-cultures of NPCs and human foreskin fibroblast. The percentage of cells immunoreactive for (A) 7-AAD, (B) nestin, PSA-NCAM, A2B5, β-tubulin III and GFAP out of the total NPC populations (n ≥ 4/group) was evaluated by ANOVA with Turkey post hoc test. Mean values ± SEM. [file jcmm0017-1434-sd2.jpg]

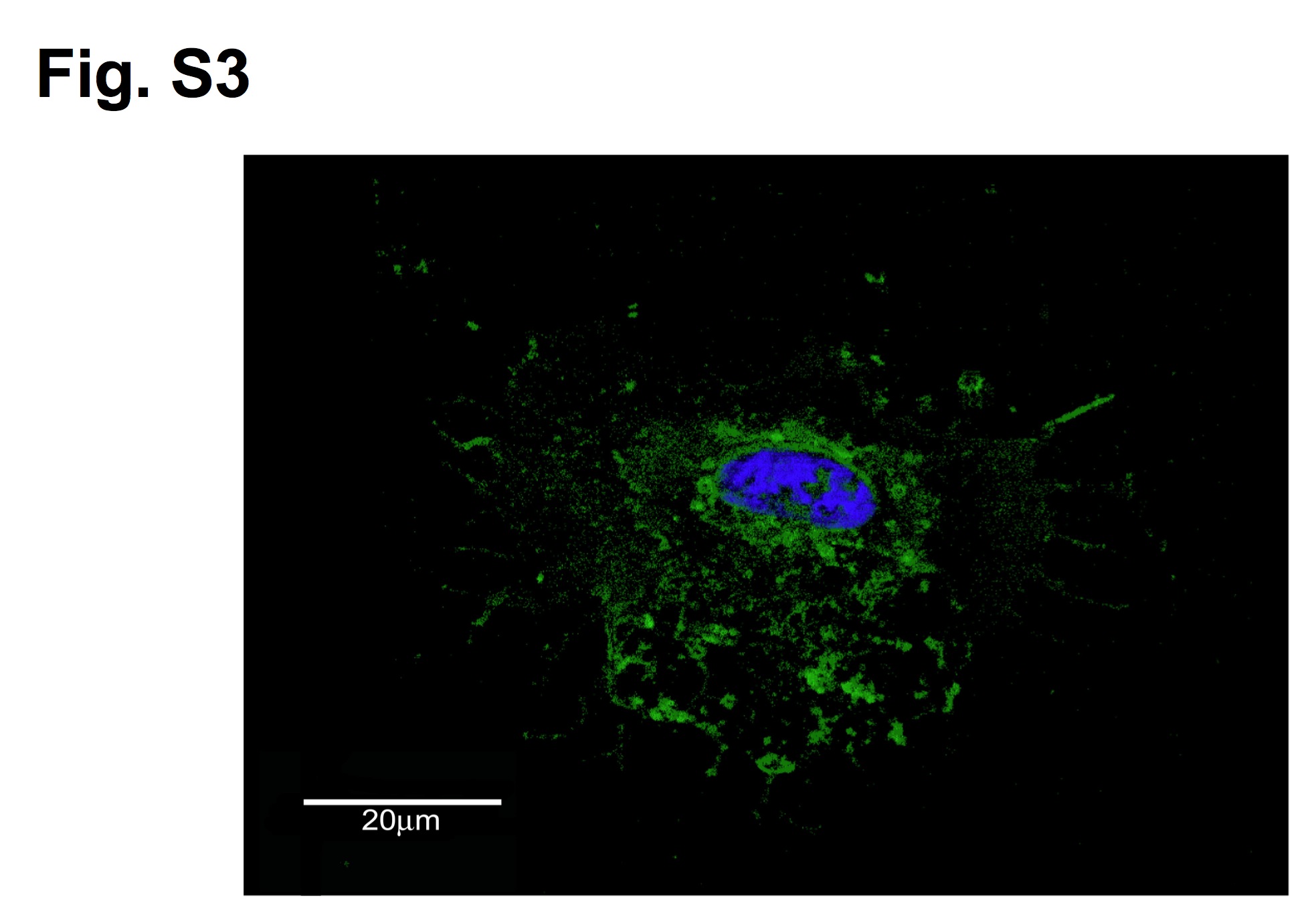

Supplement: Figure S3 — Representative confocal microphotograph of fluorescent latex beads (green) in microglia at day 7 is shown. Nuclei are labelled in blue (Hoechst). Scale bar = 20 μm. [file jcmm0017-1434-sd3.jpg]
