# Supplementary material for: Interplay between human microglia and neural stem/progenitor cells in an allogeneic co-culture model
Source: J Cell Mol Med. 2013 Sep 12;17(11):1434–43. doi: 10.1111/jcmm.12123 (PMC4117556; doi:10.1111/jcmm.12123)
Supplement: Supplementary file 4 — Table S1 Antibodies used in flow cytometry. Table S2 Antibodies used in immunocytochemistry. [file jcmm0017-1434-sd4.doc]

**Supplementary Materials**

**Supplementary Methods**

***Culture of human foreskin fibroblast***

Human foreskin fibroblasts (CRL-2429; ATCC Mananas, USA) were cultured in Iscovs DMEM medium supplemented with 10% FBS (all from Life Technologies, CA, USA) to form a confluent monolayer 37°C and 5 % CO_2_. The cell cultures were passaged once per week using TrypLE Express (Life Technologies).

***Flow cytometry***

To label cell surface markers, cells were incubated with the respective antibodies at 4°C for 30 minutes, washed, resuspended in PBS, and fixed in Cytofix fixation buffer (BD Biosciences). For intracellular staining, the cells were fixed and permeabilized by Cytofix/Cytoperm Fixation/Permeabilization Kit (BD Biosciences) following the manufacturer’s instructions prior to the incubation with the respective primary antibodies. To analyze intracellular TGF-β1 & 2, Brefeldin A (1:500, BD Biosciences) was added to the culture medium during the last 6 hours to block cytokine secretion in some experiments, followed by intracellular staining. Flow cytometry data were collected by using a FACSCalibur (Becton and Dickinson) and data analysis on percentage of immunopositive cells and their mean fluorescence intensity (MFI) was performed with the software FlowJo (Tree Star, Inc.). For detailed information on antibodies, see Supplementary Table 1.

***Immunocytochemistry***

The procedure was performed as previously described [13]. The cells cultured on cover slips were fixed with 4% paraformaldehyde for 15 minutes at room temperature, rinsed in PBS, pre-incubated with 1% bovine serum albumin and 10% normal goat serum for 2 hours, incubated with the primary antibodies overnight at 4°C and rinsed in PBS 3×10 minutes. The secondary antibodies were added for 1 hour at room temperature, followed by a rinse in PBS 3 times. For detailed information on antibodies, see Supplementary Table 2. The cell nuclei were stained with Hoechst 33342 (1:400, Life technologies) prior to mounting with the coverslips in PVA-DABCO (9.6% polyvinyl alcohol, 24% glycerol, and 2.5% 1,4-diazabicyclo (2.2.2) octane in 67 mM Tris-HCl, pH 8.0). The z-stack images were acquired using an inverted microscope Axiovert 200M (Carl Zeiss MicroImaging GmbH, Jena, Germany) connected to a LSM510 META confocal unit.

***Enzyme-linked immunosorbent assay (ELISA)***

The ELISA reagents and components for TNF-α, IL-6, IL-10, TGF-β1, TGF-β2, BDNF, GDNF and NT-3 were purchased from R&D. The experimental procedure was performed according to the manufacturer’s instructions, except for using Tris-buffered saline (TBS) instead of PBS. QuantaRed fluorescent substrate (Thermoscientific, Stockholm, Sweden) was used instead of a colorimetric substrate in the assays for TNF-a, IL-10, GDNF and NT-3. The culture media from mono- and co-cultures were processed in triplicate samples. The analysis of 96-well plates was achieved in a TECAN Safire2 microplate reader (Männedorf) at 540 nm.

**Supplementary figure legends**

***Fig. S1.*** Representative phase contrast microphotographs of mono- and co-cultures at day 7 are shown. Scale bar = 200μm.

***Fig. S2.*** Flow cytometry analysis of human NPC death and phenotype in the cultures with higher density of NPCs or co-cultures of NPCs and human foreskin fibroblast. The percentage of cells immunoreactive for (A) 7-AAD, (B) nestin, PSA-NCAM, A2B5, β-tubulin III and GFAP out of the total NPC populations (n ≥ 4/group) was evaluated by ANOVA with Turkey *post hoc* test. Mean values ± SEM.

***Fig. S3.*** Representative confocal microphotograph of fluorescent latex beads (green) in microglia at day 7 is shown. Nuclei are labeled in blue (Hoechst). Scale bar = 20μm.

**Supplementary Table 1** Antibodies used in flow cytometry

| **Conjugated antibodies** | | | | |
| --- | --- | --- | --- | --- |
| **Antibodies** | **Fluorescence conjugated** | **Clone** | | **Source** |
| CD40 | FITC | 5C3 | | BD Biosciences, San Diego, CA, USA |
| CD80 | FITC | L307.4 | | BD Biosciences |
| CD86 | FITC | 2331 | | BD Biosciences |
| CD163 | Alexa Fluor 647 | RM3/1 | | Biolegend, San Diego, CA, USA |
| CD200 | PerCP-Cy5.5 | MRC OX-104 | | BD Biosciences |
| CD200R | PE | OX-108 | | Biolegend |
| CD206 | Alexa Fluor 647 | 15-2 | | Biolegend |
| HLA-II | FITC | CR3/43 | | Dako, Glostrup, Denmark |
| PSA-NCAM | PE | 2-2B | | Miltenyi Biotec, Auburn, CA, USA |
| A2B5 | PE | 105-HB29 | | Miltenyi Biotec |
| Nestin | PerCP-Cy5.5 | aa. 402-604 | | BD Biosciences |
| GFAP | PE | Cow spinal cord homogenate | | BD Biosciences |
| β-tubulin III | Alexa Fluor 647 | Rat brain microtubules | | BD Biosciences |
| Active Caspase-3 | PE | C92-605 | | BD Biosciences |
| 7-AAD |  |  | | BD Biosciences |
| TGF-β1 | PE | 9016 | | R&D |
| **Unconjugated primary antibodies** | | | | |
| **Antibodies** | **Isotype** | **Concentration** | | **Source** |
| TGF-β2 | Rabbit IgG | 1:200 | | R&D |
| **Unconjugated secondary antibodies** | | | | |
| **Antibodies** | **Concentration** | | **Source** | |
| Anti-rabbit FITC IgG | 1:200 | | BD Biosciences | |
| **Isotype antibodies** | | | | |
| **Isotype** | **Fluorescence conjugated** | | **Source** | |
| IgM | PE | | Miltenyi Biotec | |
| IgG | FITC/PE/PerCP-Cy5.5/Alexa Fluor647 | | BD Biosciences | |

**Supplementary Table 2** Antibodies used in immunocytochemistry

| **Primary antibodies** | | | |
| --- | --- | --- | --- |
| **Antibodies** | **Species** | **Concentration** | **Source** |
| Nestin | Mouse | 1:200 | Millipore-Merck |
| β-tubulin III | Mouse | 1:800 | Covance, Princeton, NJ, USA |
| GFAP | Mouse | 1:50 | Abcam, Cambridge, UK |
| Iba-1 | Rabbit | 1:200 | Wako, Tokyo, Japan |
| **Secondary antibodies** | | | |
| Anti-mouse Alexa Fluor488 | Goat | 1:1200 | Life Technologies |
| Anti-rabbit Cy3 | Goat | 1:2400 | Jackson ImmunoResearch Laboratories, Newmarket, Suffolk, UK |
